# Genetic Pattern, Orthodontic and Surgical Management of Multiple Supplementary Impacted Teeth in a Rare, Cleidocranial Dysplasia Patient: A Case Report

**DOI:** 10.3390/medicina57121350

**Published:** 2021-12-10

**Authors:** Alessio Danilo Inchingolo, Assunta Patano, Giovanni Coloccia, Sabino Ceci, Angelo Michele Inchingolo, Grazia Marinelli, Giuseppina Malcangi, Valentina Montenegro, Claudia Laudadio, Giulia Palmieri, Ioana Roxana Bordea, Emanuela Ponzi, Paola Orsini, Romina Ficarella, Antonio Scarano, Felice Lorusso, Gianna Dipalma, Massimo Corsalini, Mattia Gentile, Daniela Di Venere, Francesco Inchingolo

**Affiliations:** 1 Department of Interdisciplinary Medicine, University of Bari “Aldo Moro”, 70124 Bari, Italy; ad.inchingolo@libero.it (A.D.I.); assuntapatano@gmail.com (A.P.); giovanni.coloccia@gmail.com (G.C.); s.ceci@studenti.uniba.it (S.C.); angeloinchingolo@gmail.com (A.M.I.); graziamarinelli@live.it (G.M.); giuseppinamalcangi@libero.it (G.M.); valentinamontenegro@libero.it (V.M.); c.lauda@hotmail.it (C.L.); giuliapalmieri13@gmail.com (G.P.); giannadipalma@tiscali.it (G.D.); massimo.corsalini@uniba.it (M.C.); daniela.divenere@uniba.it (D.D.V.); 2 Department of Oral Rehabilitation, Faculty of Dentistry, Iuliu Hațieganu University of Medicine and Pharmacy, 400012 Cluj-Napoca, Romania; 3 Medical Genetics Unit, Department of Human Reproductive Medicine, ASL Bari, 70121 Bari, Italy; emanuela.ponzi@asl.bari.it (E.P.); paola.orsini@asl.bari.it (P.O.); romina.ficarella@asl.bari.it (R.F.); mattia.gentile@asl.bari.it (M.G.); 4 Department of Innovative Technologies in Medicine and Dentistry, University of Chieti-Pescara, 66100 Chieti, Italy; ascarano@unich.it

**Keywords:** case report, cleidocranial dysplasia, supplementary teeth, supernumerary teeth, surgical extraction, genetic alteration, orthodontic and surgical management

## Abstract

*Background*: Cleidocranial dysplasia (CCD) is a rare, autosomal dominant skeletal dysplasia with a prevalence of one per million births. The main causes of CCD are mutations in the core-binding factor alpha-1 (CBFA1) or runt-related transcription factor-2 (RUNX2), located at the 6p21 chromosomal region. RUNX2 plays important roles in osteoblast differentiation, chondrocyte proliferation and differentiation, and tooth formation. The disease is characterized by clavicular aplasia or hypoplasia, Wormian bones, delayed closure of cranial suture, brachycephalic head, maxillary deficiency, retention of primary teeth, inclusion of permanent teeth, and multiple supernumerary teeth. *Materials and Methods*: A 22-year-old girl suffering from cleidocranial dysplasia with short stature, narrow shoulders, craniofacial manifestations (short face, broad forehead, etc.) and dental anomalies (different lower dental elements under eruption, supernumerary and impacted multiple teeth, etc.) was examined at our service (Complex Operative Unit of Odontostomatology of Policlinico of Bari). RX Orthopantomography (OPG) and cone beam computed tomography (CBCT) were requested to better assess the position of the supernumerary teeth and their relationships with others and to evaluate the bone tissue. *Results*: Under eruption was probably caused by dental interferences with supernumerary teeth; hence, extractions of supernumerary upper canines and lower premolars were performed under general anaesthesia. Surgery outcome was excellent with good tissue healing and improvements in the therapeutic possibilities with future orthodontics. *Conclusions:* The objective of this article is to give an update about radiological, clinical, and molecular features of CCD and to alert the health team about the importance of establishing an early diagnosis and an appropriate treatment in these patients to prevent impacted teeth complications and to offer them a better quality of life.

## 1. Introduction

Cleidocranial dysplasia (CCD) (OMIM#119600), also known as Marie and Sainton disease, is a fully penetrant, autosomal dominant genetic disorder, characterized by a large and intrafamilial clinical variability [1]. The estimated prevalence of CCD is one per million births and there is no sex predilection [2,3]. It represents a clinical continuum ranging from classic CCD, characterized by the triad of delayed closure of cranial suture, hypoplastic or aplastic clavicles, and dental anomalies, to mild forms with isolated dental anomalies in the absence of skeletal abnormalities [4]. The spectrum of anomalies is wide, ranging from patients affected by only dental and clavicles anomalies to individuals with severe defects in skeletal development. Moreover, the subjects affected by cleidocranial dysplasia could present many other systemic involvements such as vertebral bone defects at the cervical and thoracic tract, scoliosis/ lordosis, pelvic tract defects, supernumerary ribs, and defects of the hands, bones, and joints [2,5]. Dental anomalies include over retained deciduous teeth, delayed eruption teeth, unerupted permanent teeth, and supernumerary teeth [5,6,7]. CCD can be suspected at an early age, even during the fetal life by prenatal ultrasound examination [8,9]. Genetically, CCD is caused by a mutation in the osteoblast-specific transcription-factor-encoding gene, RUNX2, localised on Chromosome 6p21, which comprises a region size of 223 kb (Chr6:45328317-45551082) and consists of eight exons [10,11,12,13]. Chromosome 6p21 is responsible for the morphogenesis of the skeleton and also in the differentiation of the specific cells as osteoblasts [14,15,16]. RUNX2 is crucial for the proliferation of specific cells specialized for the synthetization of bone and tooth and also the proliferation of osteoblasts [17,18,19,20]. Runt-domain mutations are related to important dental anomalies such as supernumerary teeth, eruption alteration, etc. [21,22].

The craniofacial morphologic features of this case present a facial appearance that is characterized by a bulky forehead, hypertelorism, hypoplasia of the midface, along with other characteristics such as small or absent maxillary sinus, a thin or discontinuous zygomatic arch presenting a downward bend, and a distal curvature of the coronoid process of the mandible [3,23,24,25,26].

CCD is commonly diagnosticated at birth, but it can be missed because it has a low rate of incidence and the signs and symptoms are seldom seen [27,28].

Due to the different and complex dental anomalies, patients require the intervention of a multidisciplinary team of specialists, such as pedodontists, orthodontists, maxillofacial and oral surgeons, periodontists, and prosthodontists. With this approach, it is easy to intercept early and correct eruption alterations and malocclusions in order to promote a harmonic dentoalveolar development [3,23,28,29,30,31,32,33,34,35,36,37,38,39,40]

The aim of this article is to report the clinical and radiological findings of an adult woman diagnosed with CCD with six supplementary impacted teeth. The position and morphology of the supplementary mandibular teeth were unusual, and they likely interfered with the eruption of other dental elements. Our report also comprises the description of the procedure of the surgical extraction of the supernumerary teeth.

## 2. Clinical Report

**Figure 1:**
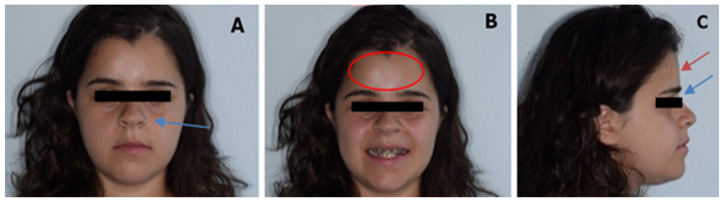
(**A**) Frontal photo, (**B**) frontal smiling photo, (**C**) lateral photo. Broad forehead and flat frontal bone (circle (**B**) and arrow (**C**)), depressed nasal bridge (blue arrow (**C**)) and wide alar base (blue arrow (**A**)).

A 22-year-old patient was referred to the Complex Operative Unit of Odontostomatology of Policlinico of Bari with the clinical diagnosis of CCD. Her father suffered from the same medical condition. Genetic analysis had never been performed, thus we requested it. Physical examination revealed short stature and narrow shoulders that she was able to press together in front of the sternum. The facial examination showed a short face, broad forehead, depressed nasal bridge, wide alar base, maxillary hypoplasia with relative mandibular prognathism, straight facial profile, and competent lips (Figure 1).

**Figure 2:**
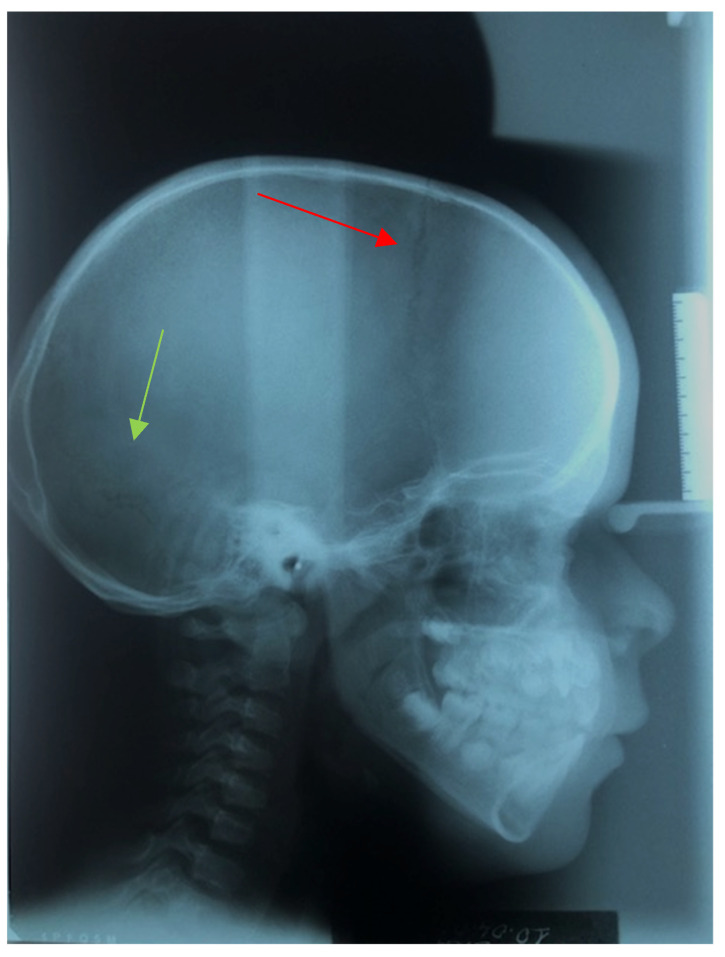
Initial 10 April 2008 teleradiography X-ray sagittal plane (10-year-old): evidence of delayed closure of cranial suture (arrows).

**Figure 3:**
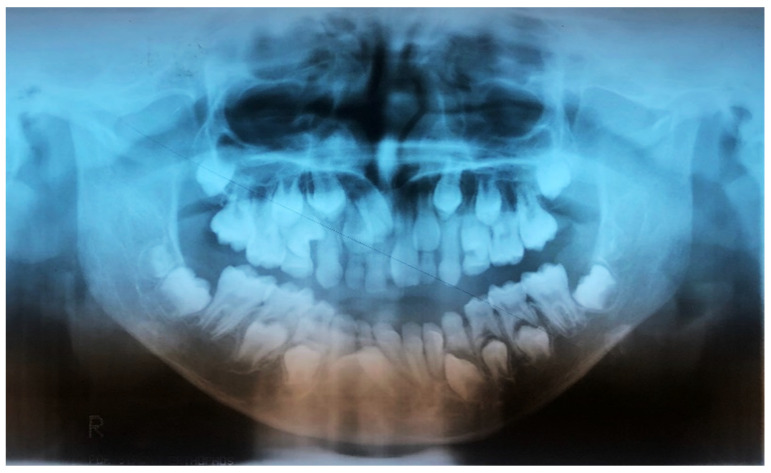
Initial 10 April 2008 orthopantomography (OPG) X-ray (10-year-old).

**Figure 4:**
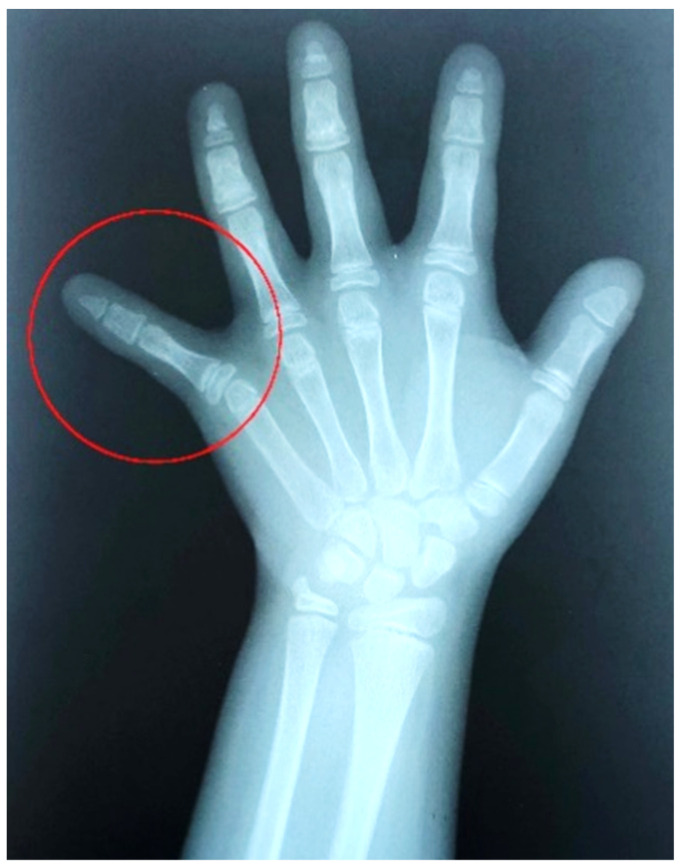
Initial 10 April 2008 left hand X-ray (10-year-old): joint alteration of the fifth finger of the left hand (red circle).

**Figure 5:**
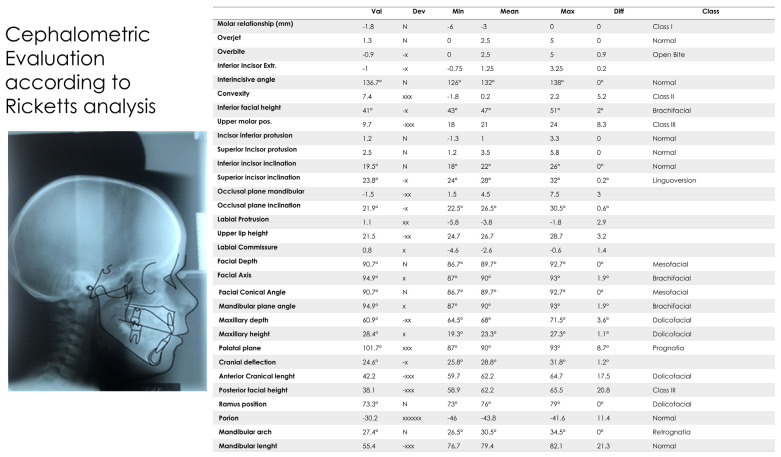
Ricketts analysis (Deltadent^®^, Outside Format—Pavia Italy) 10 April 2008.

The patient appeared to be in good health with normal intelligence. Th dental anamnesis, X-rays (Figure 2, Figure 3 and Figure 4), and cephalometric analysis (Figure 5) revealed she had been orthodontically treated in the Policlinico di Bari, Odontostomatology Division since she was 10 years old, and extractions of retained deciduous teeth had been performed to allow the eruption of permanent teeth.

**Figure 6:**
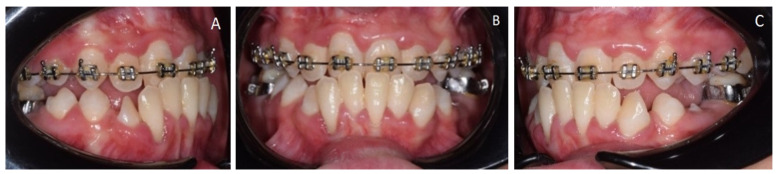
(**A**) Right occlusion, (**B**), front occlusion (**C**), left occlusion.

The maximum intercuspation (Figure 6) showed an Angle class III molar and canine relationship, an anterior crossbite caused by the premature contact of upper and lower teeth and a mandible mesial shift. The Von Spee curve was deep due to the overeruption of incisors.

**Figure 7:**
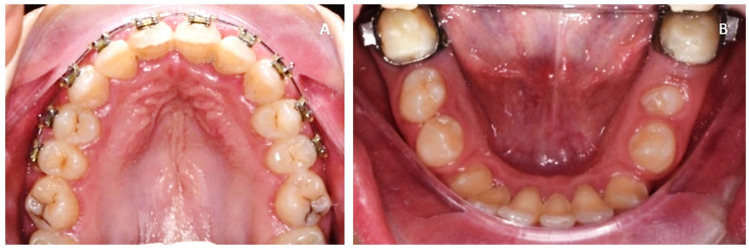
(**A**) Occlusal upper arch, (**B**) occlusal lower arch.

Oral examination of the upper arch (Figure 7A) showed: upper orthodontic multibracket appliances; contracted upper arch; 1.2 proclinate, 1.4 and 2.4 distally, 1.6 and 2.6 mesially rotated; caries of upper first molars; relevant calculus, and plaque and gingivitis on frontal teeth. Clinical examination of the lower arch (Figure 7B) showed orthodontic bands on first molars and occlusal stamps on first molars. Diastemas between 4.6 and 4.5, 4.4 and 4.3, 3.6 and 3.5, 3.4 and 3.3, crowding of the anterior segment and recessions on 3.1 and 4.2, 3.3 and 4.3 were extremely rotated, and 3.5 was under erupted.

It was suspected that the under eruption of some lower teeth was caused by interference to their natural extrusion, hence the patient underwent radiographic examination to evaluate bone and dental tissue.

**Figure 8:**
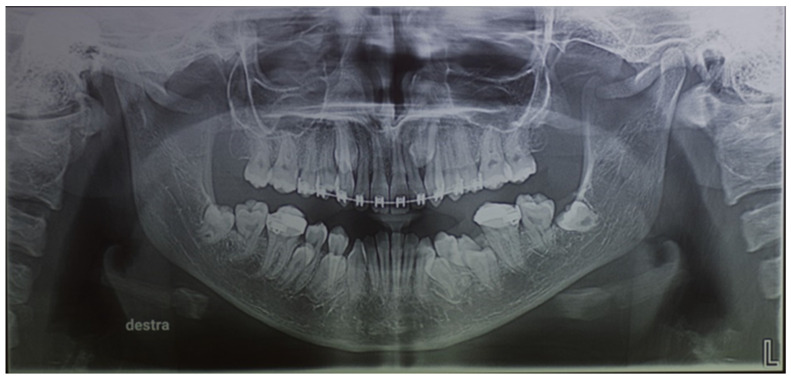
Orthopantomography (OPG) X-ray of a 21-year-old: dental laceration of 3.5, unerupted left and right lower third molar, absence of the upper third molars, and supernumerary teeth (two upper canines and four lower premolars). The impacted supernumerary teeth were immature with incomplete root development.

Orthopantomography (OPG) X-ray (Figure 8) examination showed: dental laceration of 3.5, unerupted left and right lower third molar, absence of the upper third molars and supernumerary teeth (2 upper canines and 4 lower premolars). The impacted supernumerary teeth were immature with incomplete root development.

According to the OPG, no alteration was found in the bone structure of the maxilla and mandible.

**Figure 9:**
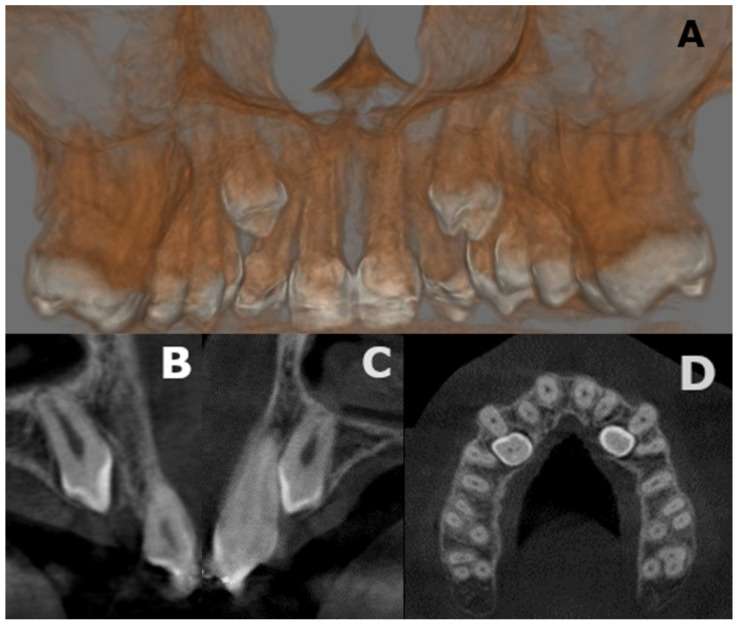
(**A**–**D**) Cone Beam Computed Tomography CBCT findings of a 21-year-old: (**A**) rendering of maxilla; (**B**) axial slice showing supplementary right canine; (**C**) axial slice showing supplementary left canine; (**D**) coronal slice. Palatal position of the upper impact teeth in zones 1.3 and 2.3.

**Figure 10:**
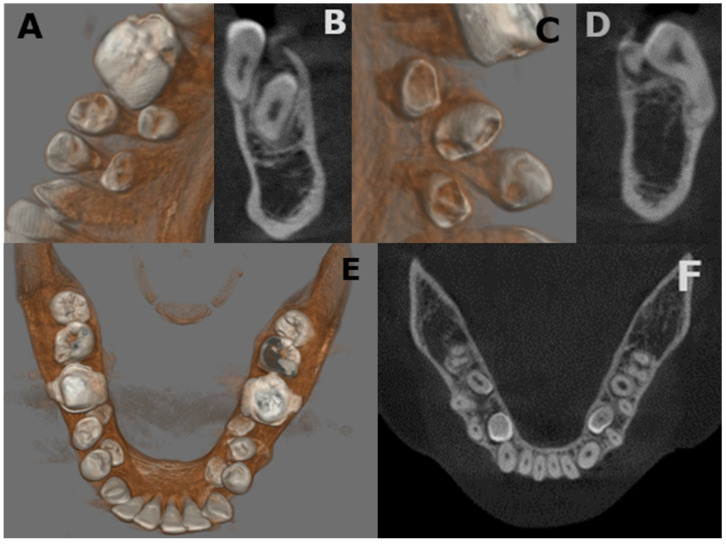
(**A**–**F**) CBCT findings of a 21-year-old: (**A**–**D**) rendering and axial slices of supplementary inferior premolars; (**E**) rendering of mandibular view; (**F**) coronal slices. Lingual position of the lower impact teeth in areas 4.4–4.5 and 3.4–3.5.

The radiological evaluation was integrated with a cone beam computed tomography (CBCT) for a better localization of the supernumerary teeth. The CBCT showed a palatal position of the upper impact teeth in zones 1.3 and 2.3 (Figure 9) and a lingual position of the lower impact teeth in areas 4.4–4.5 and 3.4–3.5 (Figure 10). Furthermore, CBCT allows us to observe the relationship between supernumerary teeth and important anatomical structures such as the mandibular nerve and the adjacent teeth apices [41].

### 2.1. Cephalometric Analysis

**Figure 11:**
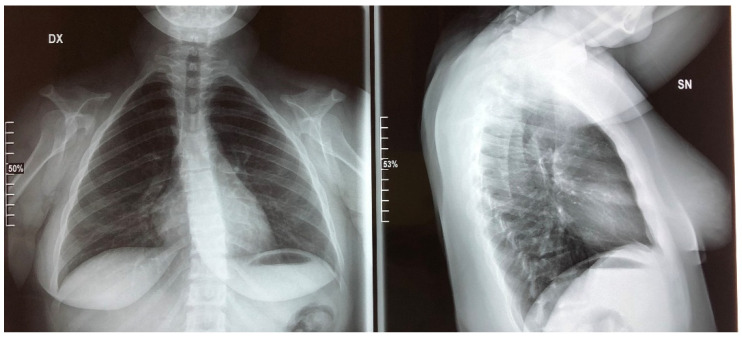
Thorax X-ray in 2021 showing a narrow and bell shape thorax.

**Figure 12:**
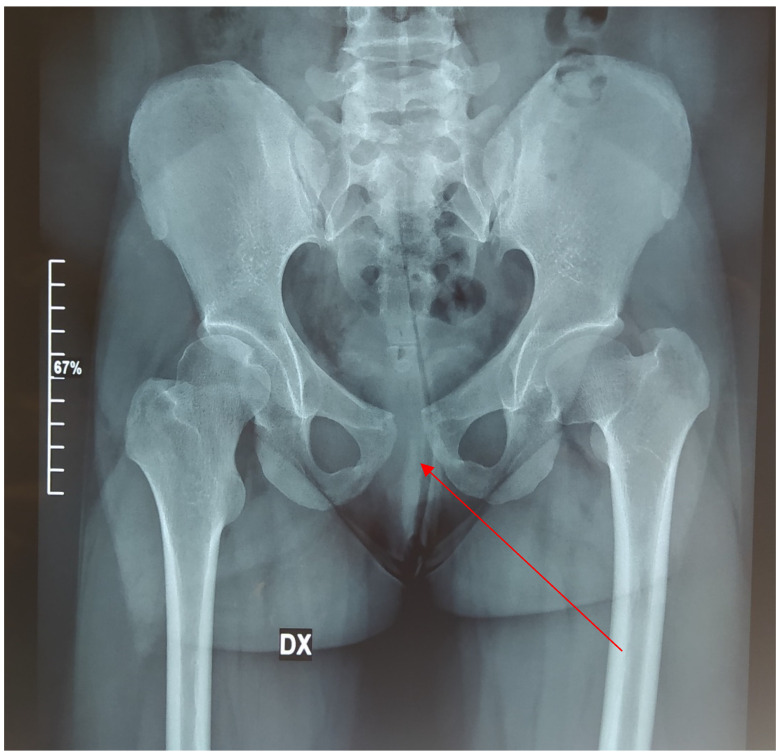
Pelvis radiography in 2021 with a wide pubic symphysis (red arrow).

**Figure 13:**
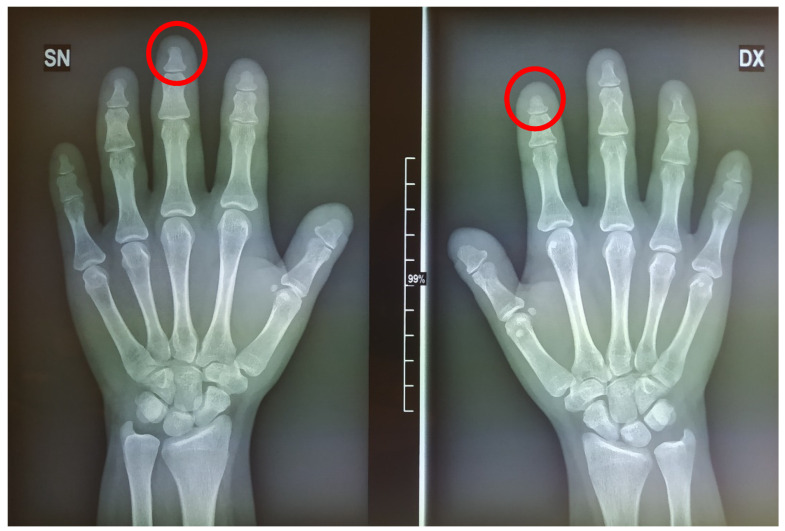
Left- and right-hand X-rays in 2021. Evident short terminal phalanges (red circles marking examples).

**Figure 14:**
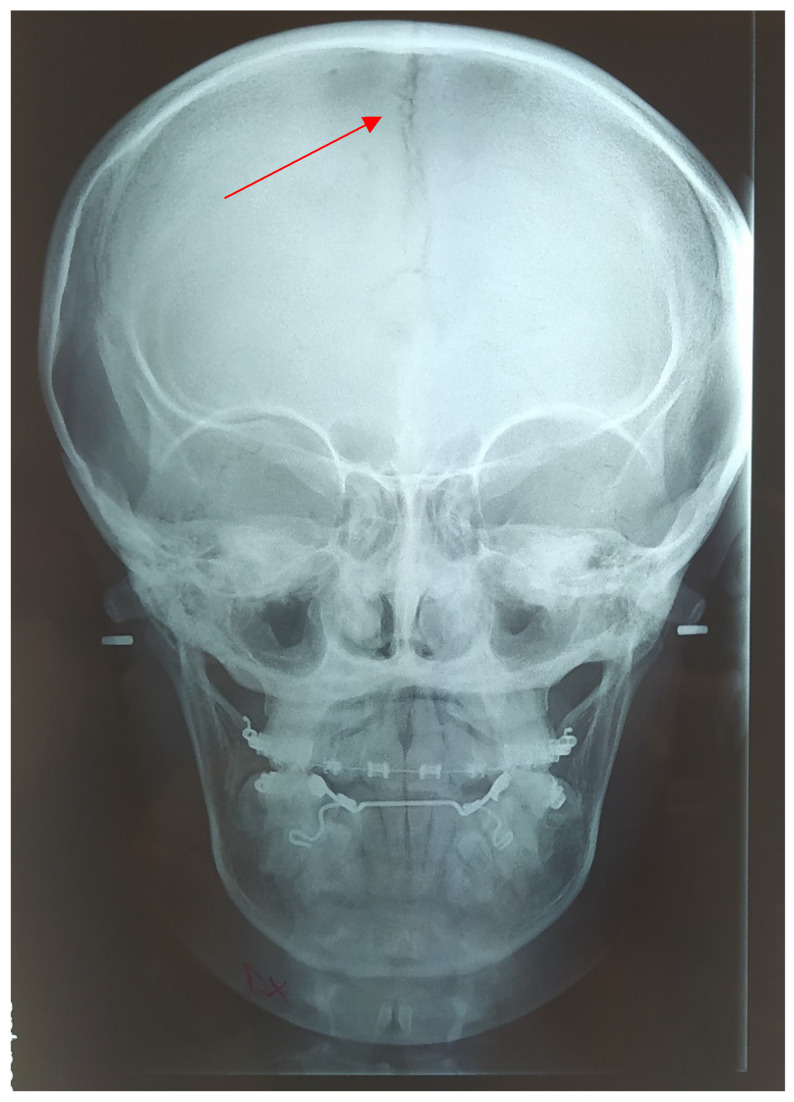
Teleradiography antero-posterior X-ray in 2021, presence of open cranial sutures (red arrow).

**Figure 15:**
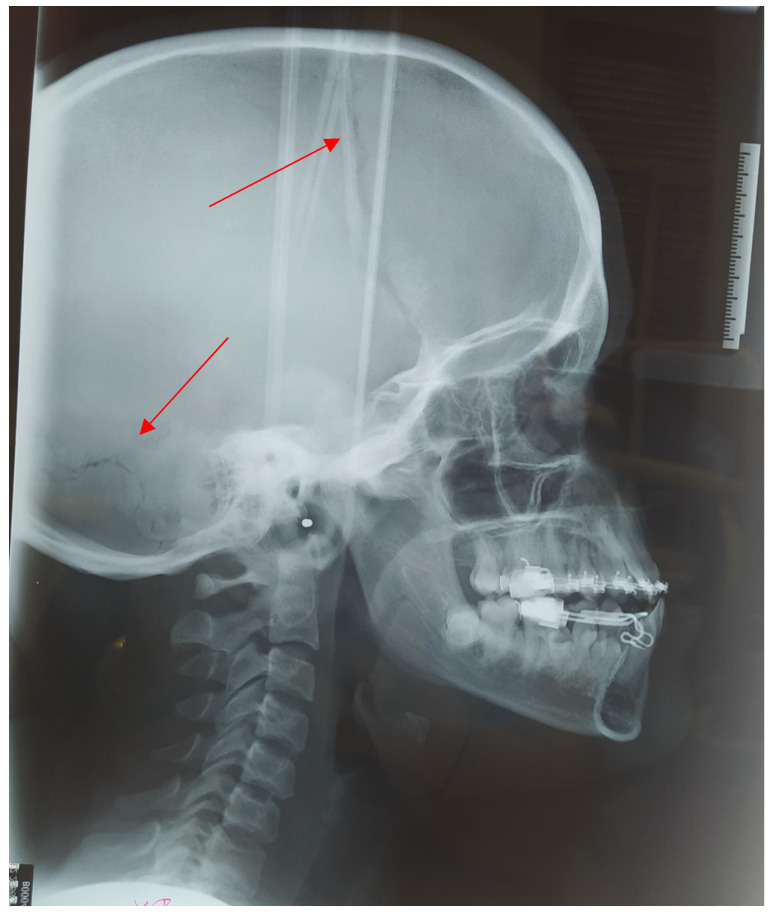
Teleradiography X-ray of sagittal plane in 2021, presence of open cranial sutures (red arrows).

**Figure 16:**
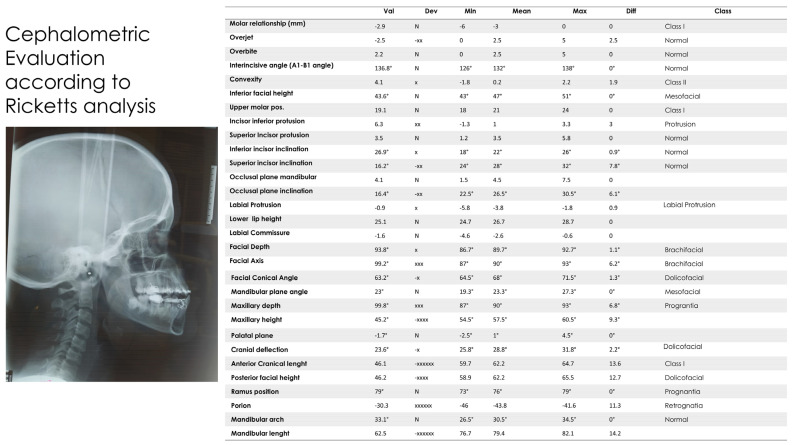
Ricketts cephalometric analysis in 2021 (Deltadent^®^, Outside Format—Pavia Italy).

In 2004, the year in which a teleradiography X-ray was performed in the laterolateral projection, the patient was 10 years old and had mixed dentition. A relevant hyperdivergency and growth of the jaw in the antero-lower direction was present. The incisors were in a head-to-head position, with overjet and overbite close to 0 [42]. According to Ricketts analysis [43,44], the position of the upper molar was in class III and overjet and overbite were very near a 0 value. The facial axis was increased, confirming the pattern of growth in hyperdivergency. The patient was in skeletal class III with retroclination of the upper incisors and normal inclination of the inferior incisors (Figure 11, Figure 12, Figure 13, Figure 14 and Figure 15) [45,46]. In 2021, according to Ricketts analysis (Figure 16), the molar relation was in dental class I, with overjet and overbite still proximal to a 0 value. Inferior facial height was augmented, and facial axis was inclined more, in particular from 94.5° to 98.9°. Skeletal class III was still relevant, and incisors were normally inclined.

### 2.2. Considerations for Supranumerary Teeth Extraction

Extraction of supernumerary teeth was necessary to allow the orthodontist to complete the treatment. In order for the patients to receive surgical treatment, they need to have good health status and an immune system that will help them recover from the surgery [47,48,49,50,51,52,53]. The difficulty in extracting the supernumerary teeth lies in the complete formation of the roots of the supernumerary elements, the almost complete absence of a bone septum between the impacted tooth and the roots of the nearby teeth, and the presence of contiguity or continuity of the impacted tooth with the inferior alveolar canal, mental nerve, and the maxillary sinus. The decision was made to proceed with the extraction of the supernumerary teeth of the 22-year-old patient in two surgical sessions under general anaesthesia; in the first operation, extractions of the upper supernumerary teeth were performed, and in the second operation, the extractions of the lower supernumerary teeth were performed.

The patient in this case report required extensive dental treatment. Indications for general anaesthesia for dental treatment include clinically compromised patients, patients with cooperation difficulties, and patients requiring extensive dental treatment [54,55].

As described by Pecci-Lloret et al. in their article, the most frequent diseases in children that require dental procedures under general anaesthesia are: encephalopathy, autism, intellectual disability, psychomotor, epilepsy, Down syndrome, etc. [56] According to Mallineni et al. central nervous system diseases and cardiovascular diseases and syndromes are all medical conditions that imply general anaesthesia when dental treatments are needed in young patients [57].

In order to be able to provide the patient with dental treatment without psychological and physical stress, it was necessary to resort to general anaesthesia, as oral surgery produces a high satisfaction score for the patient [54].

In the 24 h before the surgery, a preanaesthetic evaluation was performed, the patient was monitored with haematological tests, and an ECG evaluated the ASA grade (classification of the physical state of the American Society of Anaesthesiologists (ASA) most commonly used as a line guide for preanaesthesia assessment) [58].

In this case, the preoperative examinations did not reveal any clinical conditions worthy of note, thus it was considered suitable for the surgical procedure.

**Figure 17:**
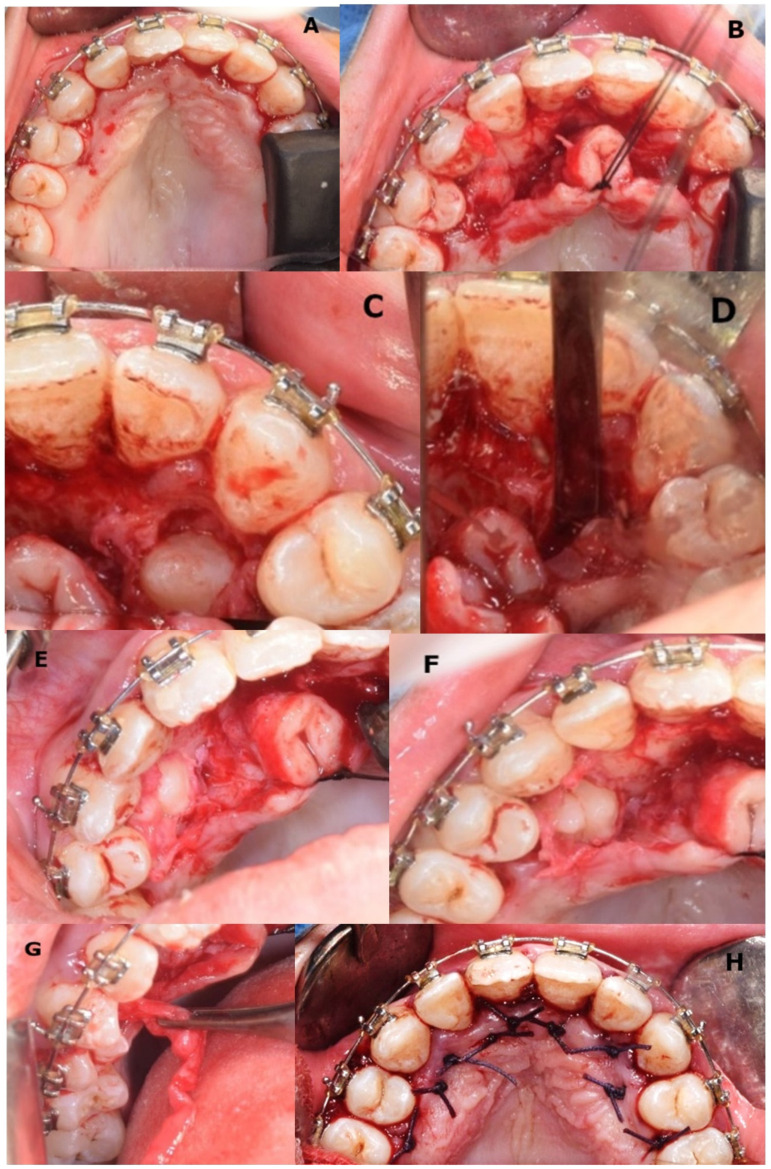
(**A**–**H**) Supplementary canine extractions 31 July 2020 (22-year-old): (**A**) full-thickness incision between mesial upper first premolars; (**B**) raising of the full-thickness flap and retention with 2-0 silk suture; (**C**) exposure of supplementary left canine; (**D**) extraction site of supplementary left canine; (**E**) partial exposure of supplementary right canine; (**F**) exposure of supplementary right canine just before extraction; (**G**) follicular sac removal; (**H**) suture of the flap.

Premedication with oral benzodiazepines was performed on the day of surgery to proceed with intravenous cannulation and sedation. Consequently, nasal intubation was performed. Patient vital signs, constantly monitored, were normal in order to avoid mild or moderate anaesthetic complications such as hypotension, airway obstruction, nausea and vomiting, and post oral surgery anisocoria [59,60,61,62]. After local infiltrative anaesthesia, a full-thickness palatal intrasulcular incision from zone 14 to zone 24 was performed with a no. 15 scalpel and the bone surrounding the canine crown was removed to expose the teeth, and a preliminary dislocation was performed. Both canines were extracted without much resistance. And the flap was repositioned and sutured with 2-0 silk (Figure 17A–H). Inferior supplementary premolars were extracted three months later. The patient underwent general anaesthesia to extract the supernumerary teeth. Given their position, it was decided for a lingual access. A lingual intrasulcular incision in regions 3.2–3.6 and 4.2–4.6 was performed using scalpel no. 15. The mucoperiosteal flap was detached and then skeletonized.

Successively, the ostectomy was performed using a round bur and the elements 3.5, 3.4, 4.4, and 4.5 were extracted using levers.

The suture with braided silk thread 2-0 was accomplished at the end. The patient presented after seven days for the removal of the sutures with good healing of the soft tissues.

### 2.3. Genetic Analysis

**Figure 18:**
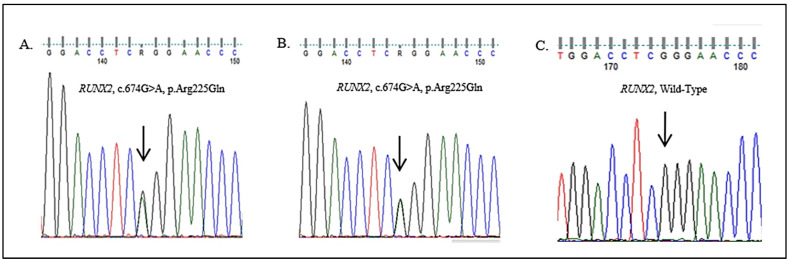
Sanger sequencing confirmation and segregation analysis of the RUNX2 mutation (NM_001024630.3:c.674G>A, NP_001019801.3:p.Arg225Gln), respectively, in the proband (**A**), the father (**B**), and the mother (**C**). The arrow points to the mutated nucleotide.

A whole exome sequencing (WES) analysis was performed using Ion Torrent Next-Generation Sequencing Platform [Ion AmpliSeqTM Exome RDY Library Preparation Kit, Ion XpressTM Barcode Adapters Kit, Ion PITM Chip Kit v3, Ion ReporterTM 5.16 Software], and data analysis revealed a heterozygous variant in the RUNX2 gene (NM_001024630.3), c.674G>A (p.Arg225Gln; ClinVar: RCV000731332; dbSNP: rs104893991) at the exon 4, an already described pathogenetic mutation. The variant was validated by Sanger sequencing (Figure 18A). The same c.674G>A variant was present in the affected father (Figure 18B).

The sequence analysis identified the following exonic variant in heterozygosity in the RUNX2 gene (NM_001024630.3), c.674G>A, which at the protein level determines the amino acid change p. Arg225GIn. The variant, potentially associated with the clinical indication of the analysis, is reported in the main databases (ClinVar: RCV000731332, dbSNP: rs104893991) and is classified as a pathogenetic variant. Pathogenetic variants in the RUNX2 gene are generally associated with cleidocranial dysplasia with autosomal dominant inheritance (cleidocranial dysplasia, CCD, OMIM # 119600). This variant was validated by Sanger sequencing, and the segregation analysis made it possible to identify the variant in the father.

The test was aimed at identifying a possible genetic cause that could explain the patient’s clinical picture. This analysis was not intended to identify variants that are outside the clinical indication of the investigation. The test does not detect duplications and deletions of one or more exons or the entire gene and low percentage mosaicisms and epimutations. It is also reported that some mutations could escape the mutational analysis, considering the intrinsic characteristics of the method (regions rich in GC and homopolyneric sequences).

## 3. Discussion

Cleidocranial dysplasia is characterized by a general dysplasia with various skeletal and dental deformities. The most important skeletal feature is clavicular aplasia or hypoplasia with consequent hypermobility of the shoulders [63] due to a defective ossification of medial and lateral clavicular centers, which are separated by a fibrocellular structure [64]. The skulls of affected individuals are characterized by brachycephaly, open cranial sutures, Wormian bones, delayed fontanelle closure, and pronounced parietal and frontal bones [1]. Facial features include hypertelorism, recession of the nasal bridge, wide alar base, and maxillary hypoplasia with relative mandibular prognathism [65,66]. The palate may be deep, and, occasionally, cleft palate may occur [66]. Affected people may develop recurrent sinus and ear infections due to abnormalities of the facial skeleton, highly arched palates, and reduced paranasal sinuses [67]. Other common characteristics are short stature, wide pubic symphysis, and short terminal phalanges. Moreover, CCD patients may present brachydactyly, genu valgus, pes planu, dysplastic scapulae, and scoliosis [3,68] (Figure 11, Figure 12, Figure 13, Figure 14 and Figure 15).

Dental anomalies are a characteristic feature in CCD. The most significant dental anomalies are multiple supernumerary teeth with retention cysts [17] that compromise the cosmetic appearance of the dentition and function [64]. They are easily revealed in OPG X-rays but also on the upper anterior occlusal X-ray and Computed Tomography Cone Beam (CBCT) [69,70,71]. In particular CBCT allows for the assessment of the precise supernumerary teeth localizations and their relationships with other teeth and important anatomical structures [72].

The cause of supernumerary teeth formation may be an incomplete or delayed resorption of dental lamina, hence remnants of the dental lamina may become reactivated to form a supernumerary tooth [73]. Other dental anomalies are retention of deciduous teeth and delayed eruption with consequent impaction of the permanent teeth. The causes of unerupted teeth include: abnormal bone resorption, decreased alkaline phosphatase levels, absence of cellular cementum and increase of acellular cementum on the affected teeth’s roots, and the interposition of fibrous tissue between the dental follicle and the mucosa acting as a barrier to eruption [74,75].

The most common sites where supernumerary teeth are present are maxillary incisors site as well as maxillary and mandibular canines and premolar areas [72]. The presence of supernumerary teeth may cause the failure of eruption of permanent teeth and the resorption of adjacent roots [64]. In our case, the supernumerary teeth were unerupted and located in the upper canine area and in the lower premolar area, hindering the eruption of 3.5. None of these teeth had retention cysts.

Other dental anomalies described in literature but not seen in this patient are enamel and cementum hypoplasia, root dilaceration, and microdontia [76].

The peculiarity of the present case report is that the patient has all the clinical characteristics of dysostosis, and, in addition to the typical characteristics of dysostosis (flat forehead, open skull fontanelles, short stature, etc.), she has more supernumerary teeth larger than the two typical teeth of dysostosis. In fact, the upper maxillary arch has two supernumerary teeth in the palatal areas of 2.3 and 1.3. In addition to these two upper supernumerary teeth, there are four more supernumeraries in the mandibular arch, specifically 4.4, 4.5, 3.4 and 3.5.

No specific therapeutic guidelines are available for dental management in CCD patients. Therapeutic plans depend on patient age, craniofacial characteristics, dental anomalies, and social and economic circumstances [3]. Management of the dental abnormalities depends on the dental and chronological age of the affected individual and requires a multidisciplinary approach with the cooperation of the orthodontist, paediatric dentist, oral surgeon, and prosthodontist [77]. Early diagnosis is important to formulate an appropriate treatment plan and to achieve a successful outcome [78]. Treatment options include removal of retained deciduous and supernumerary teeth and orthodontic traction of impacted permanent teeth [2]. In some cases, orthognathic surgery at the end of growth is indicated to establish a correct relationship between the maxillary jaws. In older patients with CCD, another possibility is prosthetic treatment which allows for the restoration of aesthetics and function in a short time [76].

Many treatments were sought in order to find the most appropriate one. Chae et al. suggested an innovative training approach for the extraction of supernumerary teeth using a three-dimensional printed model. This method improved surgical skills and, in particular, shortens the learning curve in beginners [79]. Some new techniques like the use of PRP were also tested [80,81,82].

In this case, extractions of supernumerary teeth were fundamental in order to successfully conclude the orthodontic treatment and give the patient a correct occlusion and an aesthetic smile. Extractions were planned with the aim of promoting correct eruption and subsequent alignment of the physiological dental elements. Orthognathic surgery was not considered necessary as the patient already had acceptable aesthetics.

Another peculiar aspect was related to the treatment duration. In fact, these patients require a long-term treatment and follow up until dental eruption and skeletal growth has been completed. Our patient had been treated for 12 years, since she was 10 years old, following her development pattern and deciduous teeth exfoliation.

Interestingly, genotype–phenotype correlations have been established for the dental manifestations. No clear correlation has been established between genotype and clavicular involvement [17,21,83]. The classic form of CCD is associated with heterozygous RUNX2 pathogenic variants located in the Runt domain that abolish the transactivation activity of the mutated protein with consequent haploinsufficiency [21]. Clinical features like short stature and dental anomalies seem to be milder in individuals with classic CCD in which an intact Runt domain and higher residual RUNX2 activity is present. A clinical spectrum ranging from isolated dental anomalies without skeletal features to mild and classic forms of CCD are associated with hypermorphic pathogenic variants that result in partial loss of protein function (c. 1171C>T[p.Arg391Ter], c.598A>G [p.Thr200Ala],c.90dupC). Patients with a heterozygous pathogenic frameshift variant, c.1205dupC, reflecting the role of RUNX2 protein in the maintenance of adult bone, presented with osteoporosis leading to recurrent bone fractures and scoliosis [84,85,86,87,88,89,90,91,92,93,94,95,96,97,98,99,100,101]. Osteoporosis leading to recurrent bone fractures and scoliosis has been associated with a heterozygous pathogenic frameshift variant, c.1205dupC, reflecting the role of RUNX2 protein in the maintenance of adult bone [102].

Skeletal dysplasia are genetic diseases of chronic evolution and they require multidisciplinary treatment, focused on measures of symptomatic support but also preventive and pre-emptive [63].

Other conditions share some characteristics with CCD spectrum disorder. The fact that similar skeletal elements are affected suggests that some of these conditions may result from mutations of genes that affect the action of RUNX2 on its downstream targets. Most notable is the association with the 16q22.1 deletion that includes the characteristics of CBFB with wide-open fontanelles and short clavicles [103]. Because CBFB forms a heterodimer with RUNX2 to activate transcription of downstream targets, CBFB haploinsufficiency would explain the similarity in the phenotypes. Other disorders to consider in the Differential Diagnosis of Cleidocranial Dysplasia (CCD) Spectrum Disorder are: mandibuloacral dysplasia (OMIM PS248370) [LMNA, ZMPSTE24 gene], pycnodysostosis [CTSK], Yunis Varon syndrome (OMIM 216340) [FIG4], hypophosphatasia [ALPL], and parietal foramina with cleidocranial dysplasia [MSX2]. Although the clinical characteristics of the patient guided the diagnosis towards cleidocranial dysplasia, scientific evidence of other disorders entering into differential diagnosis with cleidocranial dysplasia identified whole exome sequencing as a cost effective gene test compared to Sanger Sequencing.

In our case, the c.674G>A mutation is located in exon 4 and the correspondent amino acid change R225Q is located in the Runt domain. It results in haploinsufficiency of RUNX2 and poor cellular differentiation of osteoblastic precursors. The affected arginine residue is located at the c-terminal region of the Runt domain, is highly conserved in different species, and the resulting amino acid change interferes with nuclear localization and DNA binding of the RUNX2 protein as reported in previous studies [85,86,87,91,92,93], affecting the osteoblast differentiation and skeletal morphogenesis, due to its role as the master transcriptional factor involved in bone formation.

Clinical features like short stature and dental anomalies seem to be milder in individuals with classic CCD in which an intact Runt domain and higher residual RUNX2 activity is present [88].

RUNX2 presents two in-frame ATG codons, both of which can serve as potential translation start sites, and both promoters drive expression of both isoforms with different spatiotemporal patterns, which suggests specialized functions of each [89]. The gene expression from the proximal promoter (P2) generates type I RUNX2 mRNA differing at the 5′ end from the type II RUNX2 mRNA being under the control of the P1 distal promoter. P1 promoter is termed ‘bone-related’ because of the driving expression of the isoform widely associated with bones [90,91,92,93,94]. The protein, runt-related transcription factor 2 (RUNX2), is a transcription factor involved in osteoblast differentiation and skeletal morphogenesis [95]. In detail, RUNX2 type I and type II regulate expression of bone-related genes; however, it has been suggested that they might have different functions in skeletogenesis [96]. Type I is expressed in T cells, osteoblasts, and chondrocytes [97] and contributes to the intramembranous bone development [98]. The expression of Type II increases during osteoblast differentiation [92,99], and it has been shown to be responsible for endochondral bone formation [89]. Moreover, RUNX2 is also responsible for regulating expression of genes that, when deregulated, cause craniosynostosis like NEL-like 1 (NELL1) [100]. Since it was suggested that RUNX2 regulates not only skeleton development but also the expression of mesenchymal tissue, controlling differentiation of dental epithelium, it could partially explain dental abnormalities. A study on the cellular mechanism of tooth eruption showed that the eruption of teeth in heterozygous Runx2/Cbfa1 knockout mice was significantly delayed compared with wild type mice due to the impaired recruitment of osteoclasts [101].

Mutations associated with severe dental abnormalities (supernumerary teeth, eruption failure) affect the Runt domain. In contrast, mild dental problems are correlated with mutations outside the Runt domain [17,78,88].

## 4. Conclusions

Cleidocranial dysplasia is a rare disease in our country and environment. It comprises a special, visible, and noticeable clinical pattern, especially if a thorough physical examination is performed on the patient at an early stage. It is important to prevent complications associated with dental impacted teeth with early dental diagnosis and early multidisciplinary management. Most of the cases do not have a specific treatment, but surgical interventions could be done under certain circumstances to correct physical anomalies and, thus, improve the quality of life of patients.

## Data Availability

All experimental data to support the findings of this study are available contacting the corresponding author upon request. The authors have annotated the entire data building process and empirical techniques presented in the paper.
